# Lenticulostriate arteries appearance before thrombectomy predicts good outcome in acute middle cerebral artery occlusion

**DOI:** 10.1186/s12883-020-01716-1

**Published:** 2020-04-16

**Authors:** Feifeng Liu, Chen Chen, Lan Hong, Hao Shen, Wenjie Cao, Qiang Dong, Xinyi Yang, Mengruo Guo, Ying Li, Yaping Xiao, Xin Cheng, Gang Li

**Affiliations:** 1 Department of Neurology, Shanghai East Hospital, Tongji University, Shanghai, China; 2 Department of Neurology, Shanghai huashan hospital, Fudan University, Shanghai, China

**Keywords:** Middle cerebral artery occlusion, Lenticulostriate artery, Thrombectomy, Outcome, Ischemic stroke

## Abstract

**Background:**

Endovascular therapy is widely used in acute large vessel occlusion. This study investigated whether imaging of lateral lenticulostriate arteries (LSAs) before thrombectomy would potentially be helpful for predicting prognosis of patients with acute M1 segment of middle cerebral artery occlusion (MCAO).

**Methods:**

59 consecutive patients with acute M1 segment of MCAO treated with mechanical thrombectomy at two comprehensive stroke centers were analyzed. Patients were categorized into LSA+ (appearing of lateral LSAs) and LSA- (sparing of lateral LSAs) group according to preprocedural digital substraction angiography (DSA). Baseline data and clinical outcomes were compared. A good clinical outcome was defined as a modified Rankin Scale score of 0 to 2 at 3 months. The association between clinical and imaging parameters and functional outcome was evaluated with logistic regression analysis.

**Results:**

LSA+ was shown in 36 patients (61%). LSA+ group had a significantly higher proportion of good outcome (72.2% vs. 8.7%, OR 27.3,95% CI 5.38–138.4, *P* < 0.001), lower risk of symptomatic intracranial haemorrhages (sICH) (8.3% vs. 47.8%,OR 0.10,95% CI 0.02–0.42, *P* = 0.001) and lower mortality in hospital (5.6% vs. 34.8%, OR 0.11,95% CI 0.02–0.58, *P* < 0.004) compared with LSA- group. Patients in LSA+ group had lower baseline NIHSS score(*P* < 0.01) and NIHSS score at 14 days(*P* < 0.01) and smaller infarct core volume (*P* = 0.016) on computed tomography perfusion imaging (CTP) compared to the LSA- group. Multivariate logistic regression analysis showed that a small infarct core volume (OR 6.74,95% CI 1.148–39.569, *P* = 0.035) and LSA+(OR 22.114,95% CI 3.339–146.470, *P* = 0.001) were associated with a good clinical outcome.

**Conclusions:**

Our data suggest that appearance of lateral LSAs before mechanical thrombectomy would be potentially helpful for predicting favorable prognosis of patients with acute M1 segment of MCAO.

## Background

Randomized trials have demonstrated that second-generation endovascular thrombectomy had an obvious clinical benefit over medical therapy alone among patients with emergent large artery occlusion (LAO) [1–5]. However, poor clinical outcomes despite successful revascularization are still common [6]. Thus, patient selection and prognosis prediction are still key issues for thrombectomy. In addition to the time to treatment, infarct core volume, penumbra and collateral circulation, more concise imaging parameters are worth exploring [7–9]. It is known that collateral circulation and time from onset to treatment both affect the transformation from penumbra to infarct core [7, 8]. However, the area supplied by lateral lenticulostriate arteries (LSAs) lacking of collateral vessels may play a unique role in acute middle cerebral artery occlusion (MCAO) independent of time and collateral flow. Thus, the objective of this study was to investigate whether the lateral LSAs observed before thrombectomy could predict good clinical outcomes.

## Methods

### Patients

From August 2016 to August 2018, we retrospectively collected all the patients presented with acute stroke due to acute M1 segment of middle cerebral artery occlusion treated with stent-retriever thrombectomy as the first-line endovascular therapy at two comprehensive regional stroke centers, Shanghai East Hospital and Huashan Hospital. All patients underwent an initial imaging protocol including nonenhanced computed tomographic (CT) scan, CT perfusion (CTP) and computed tomography angiography (CTA) before commencing endovascular thrombectomy procedures. CTP/CTA imaging was acquired on the 320-slice CT scanner (Aquilion One, Toshiba Medical Systems) and a 64-slice detector scanner (Discovery CT750 HD; GE Medical Systems, Waukesha, Wisconsin). The inclusion criteria were as follows: [1] acute ischemic stroke, [2] M1 segment of MCAO detected with digital subtraction angiography (DSA), [3] the time from onset to reperfusion therapy was determined by local guidelines at the time [10, 11]. Patients with poor imaging quality were excluded. The modified Rankin Scale (mRS) was assessed at 3-month through telephone by a staff who was blinded to the clinical data routinely. And this is part of daily clinical practice. The mRS score was recorded as 6 in the 3-month follow-up when the patients died in the hospital. A conventional consent to use the patients’ clinical data and a 3-month telephone follow-up for possible future research was obtained from each patient or their families during hospitalization. The study was approved by the institutional ethics committee and no additional informed consent was required based on the retrospective study design.

### Endovascular treatment and DSA assessment

Solitaire stent and manual aspiration thrombectomy were performed as the first-line endovascular treatment. When stent-retriever thrombectomy was not ideal, intra-arterial recombinant tissue plasminogen activator (rtPA) infusion or stenting was performed. Successful revascularization was defined as a modified thrombolysis in cerebral infarction (TICI) grade of 2b or 3 [12]^.^

**Fig. 1:**
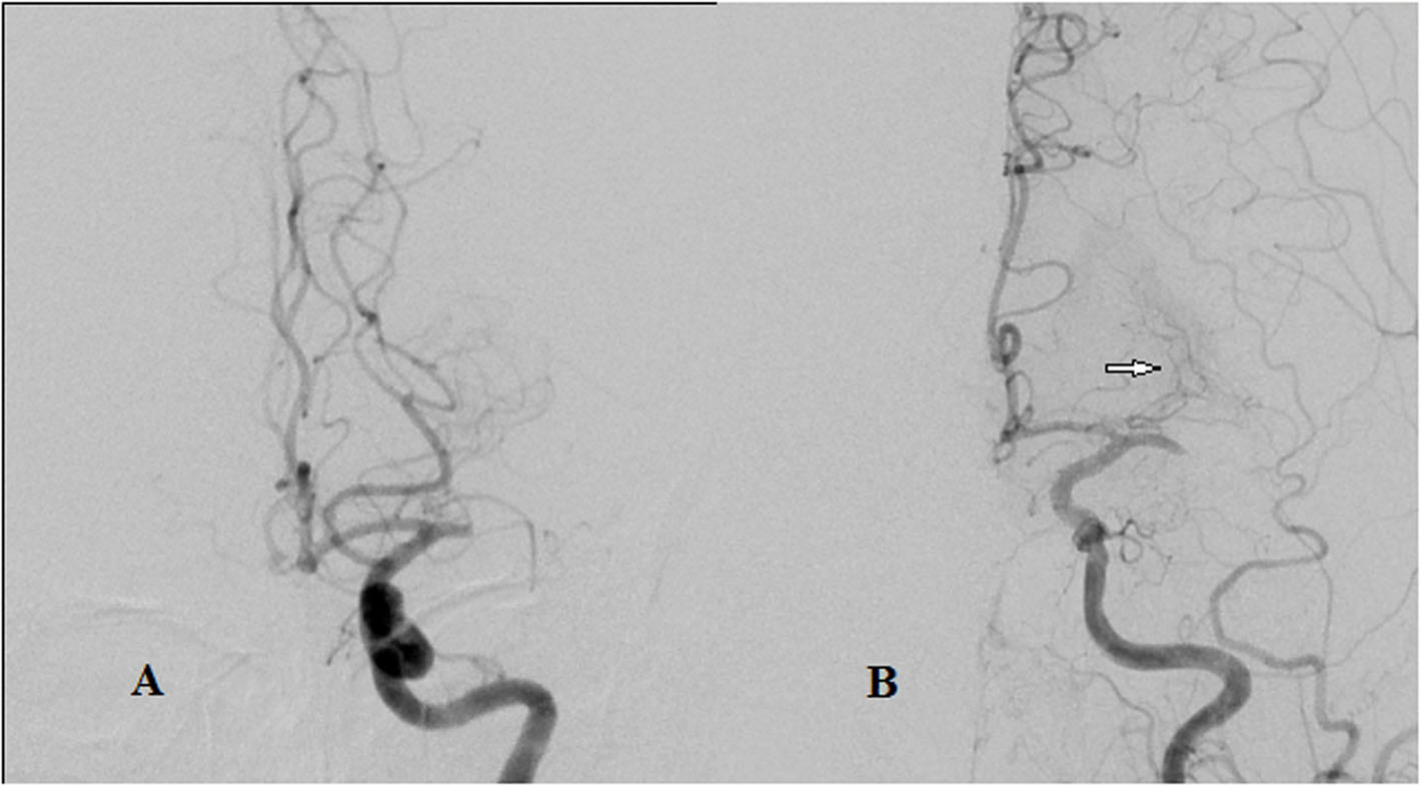
LSAs Discrimination. **a**. LSA-: No perforator artery from LMCA supplying basal ganglia on Towne’s position in DSA. **b**. LSA+: A group of lateral lenticulostriate arteries (white arrow) originating from LMCA supplying basal ganglia on Towne’s position in DSA

Two reviewers (XYP and SH) independently analyzed the pre-interventional DSA. Both readers who had at least 3 years of training in neuroradiology respectively run blinded to the interventional imaging and all clinical data. The readers determined the presence or absence of lateral LSAs on pre-interventional DSA (see Fig. 1 for illustrative cases). Each reader repeated his assessment 14 days later blinded to his first assessment in order to evaluate intra-rater reliability. Cohen’s kappa was calculated to evaluate inter- and intra-rater agreement. In cases of discrepancies, a consensus read was performed with both readers and the third more experienced reader (LG) who was not involved in the initial assessment.

### Variables and clinical assessment

Baseline data were included age, sex, cigarette smoking, medical history, time of onset, treatment and initial assessment by multi-model CT. The National Institute of Health Stroke Scale (NIHSS) score (scores range from 0 to 42, with higher scores indicating increasing severity) was evaluated before thrombectomy, and collateral status was evaluated using the regional leptomeningeal score on CTA (rLMC) and defined good collateral circulation as rLMC Score 11–20 based on previously published criteria [13].CT perfusion data were postprocessed by a commercial software (MIStar; Apollo Medical Imaging Technology, Melbourne, Australia). The infarct core was defined as relative cerebral blood flow (rCBF) < 30% and penumbra was based on the differences between the infarct core and lesion with delay time (DT) > 3 s. The mismatch ratio was defined as the proportion of DT > 3 s lesion volume (hypoperfusion) with rCBF< 30% lesion volume (infarct core) [14]. Symptomatic intracranial haemorrhage (sICH) transformation was defined according to European Cooperative Acute Stroke Study (ECASS)-III criteria [15]. The recanalization time was defined as interval between symptom onset and first intracranial DSA series. The primary outcome was defined as good outcome (modified Rankin Scale (mRS) score of 0 to 2) at 90 days. And we also evaluated core infarct volume, NIHSS(NIHSS score of 42 was assigned in cases of death) and mRS at 14 days, sICH, mortality in hospital and at 90 days, recanalization rates as secondary outcomes.

### Statistical analysis

Statistical analysis for categorical variables included Chi-square test, Fisher exact test when cell sizes were small, and odds ratios for selected comparisons. The measurement data were described as mean ± standard deviation if normally distributed or the median and analyzed using Student’s t test or the Mann-Whitney test. Logistic regression analysis was performed to identify independent predictors for clinical outcome. Variables with a *P* value of < 0.15 in the univariate analysis on clinical outcome were included in a multivariate logistic regression, performed with the forward selection and backward elimination method. A *P* value of < 0.05 was considered significant. All analytic procedures were conducted in SPSS version 21.0.

## Results

**Fig. 2:**
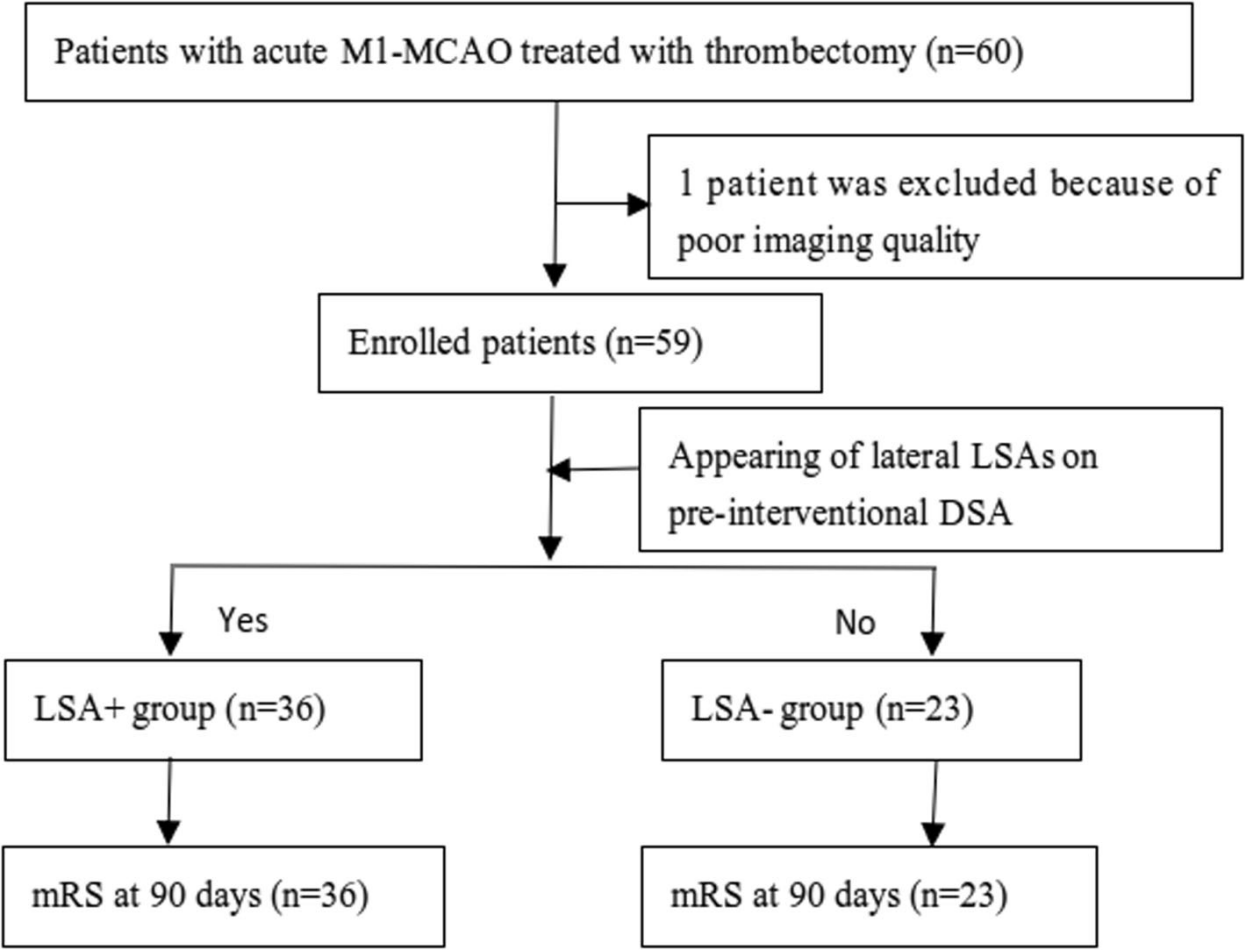
The study flow chart

We initially identified a total of 60 patients meeting the inclusion criteria. One patient was excluded because of poor imaging quality. Among the leaving 59 patients, the mean age was 69.8 ± 11.2 years, 40 patients (67.8%) were female, and the median baseline NIHSS was 15. 25 patients (42.4%) received intravenous thrombolysis treatment before thrombectomy (31 patients with contraindications and 3 failed to obtain consents which was officially acquired in China) and the mean door-to needle time (DNT) was 51 min. On pre-intervention DSA, 36 patients (61%) had the appearance of lateral LSAs (LSA+) and 23 patients (39%) were sparing of the LSAs (LSA-). There were initial disagreements on the group assignment of 2 patients, which represented 96.6% interrater agreement. Consensus was then reached on the final group assignments for these discordant designees. The interrater and intrarater reproducibility of LSAs were nearly reliable (kappa coefficient = 0.929). All 59 patients completed follow-up at 90 days. Study flow chart was summarized in Fig. 2.

### Baseline data

Baseline demographics and details of intervention were similar between groups except for NIHSS score before thrombectomy (Table 1). No significant differences were observed between the two groups in relation to age, sex, premorbid history of hypertension, diabetes mellitus, atrial fibrillation, dyslipidemia, ischemic stroke, antiplatelet drugs use, anticoagulation drugs use, cardioembolic source, history of smoking, intravenous thrombolysis, onset-to-treatment time and successful recanalization between the two groups. There was a significantly smaller ischemic core volume (34 ml versus 46 ml, *P* = 0.016) on CTP compared to the LSA- group. NIHSS score of LSA+ group was significantly lower than that of LSA- group. (14 versus 16, *P* = 0.004) (Table 1).

**Table 1:** Demographics and Aspects of the Intervention Between Patients LSA+ and LSA- Patterns

Characteristic	LSA+(*n* = 36)	LSA-(*n* = 23)	All patients(*n* = 59)	*p* value
Age,years,mean ± SD	69.3 ± 11.2	70.6 ± 11.4	69.8 ± 11.2	0.342
Gender,female(%)	24 (66.7)	16 (69.6)	40 (67.8)	0.816
Premorbid history of: n (%)				
Hypertension	17 (47.2)	13 (56.5)	30 (50.8)	0.486
Diabetes Mellitus	5 (13.9)	5 (21.7)	10 (16.9)	0.433
Dyslipidemia	4 (11.1)	3 (13.0)	7 (11.9)	0.823
Ischemic Stroke	11 (30.6)	5 (21.7)	16 (27.1)	0.458
Cardioembolic Source, n(%)	18 (50.0)	12 (52.2)	30 (50.8)	0.871
Smoking, n(%)	10 (27.8)	7 (30.4)	17 (28.8)	0.826
Antiplatelet, n(%)	10 (27.8)	6 (26.1)	16 (27.1)	0.877
Anticoagulation, n(%)	5 (13.9)	4 (17.4)	9 (15.3)	0.715
Intravenous rtPA, n (%)	13 (36.1)	12 (52.2)	25 (42.4)	0.223
DNT, min, mean ± SD, (n)	54 ± 25(*n* = 13)	49 ± 18(*n* = 12)	51 ± 21(*n* = 25)	0.217
NIHSS before thrombectomy, median (IQR)	14 (10–17)	16 (15,20)	15 (14–18)	0.004^**^
Onset to CTP time, min, median (IQR)	186 (112–295)	134 (101–189)	160 (108–257)	0.103
Onset to puncture time, min, median (IQR)	320 (227–448)	255 (180–305)	264 (214–402)	0.069
Onset to clot first reperfusion time, min, median (IQR)	357 (280–484)	305 (189–347)	316 (280–445)	0.148
LMCA occlusion, n(%)	21 (58.3)	18 (78.3)	39 (66.1)	0.115
Local anesthesia, n(%)	28 (77.8)	18 (78.3)	46 (78.0)	0.965
Successful recanalization (TICI 2b/TICI 3), n (%)	33 (91.7)	18 (78.3)	51 (86.4)	0.142
Ischemic core volume, ml, mean ± SD	34 ± 23	46 ± 41	48 ± 31	0.016^*^
Mismatch ratio, median (IQR)	3.2 (2.6–4.5)	3.5 (2.7–4.4)	3.3 (2.6–4.4)	0.625
rLMC Score, median (IQR)	14 (12–16)	14 (10–16)	14 (12–14)	0.391

Note: *rtPA* recombinant tissue plasminogen activator, *LMCA* left middle cerebral artery; mismatch ratio: hypoperfusion/infarct core;rLMC Score:the regional leptomeningeal score. *:*P* < 0.05;**:*p* < 0.01

### Clinical outcome

As primary outcome, the proportion of patients with a modified Rankin score of 0 to 2 at 90 days was 72.2% in the LSA+ group and 8.7% in the LSA- group (OR 27.3,95% CI 5.38–138.4, *P* < 0.001, Table 2). When compared to the LSA- group, the LSA+ group had a lower risk of symptomatic intracranial haemorrhages (sICH) (8.3% versus 47.8%,OR 0.10,95% CI 0.02–0.42, *P* = 0.001) and mortality in hospital (5.6% versus 34.8%, OR 0.11,95% CI 0.02–0.58, *P* < 0.004). Patients in LSA+ group had lower NIHSS (2 versus 15, *P* < 0.001) and mRS (2 versus 5, *P* < 0.01) at 14 days.

**Table 2:** Clinical Outcomes of patients Between Patients LSA+ and LSA- Patterns

Characteristic	LSA+(*n* = 36)	LSA-(*n* = 23)	*p* value	OR(95%CI)
sICH,n(%)	3 (8.3)	11 (47.8)	0.001	0.10 (0.02–0.42)
Death in hospital,n(%)	2 (5.6)	8 (34.8)	0.004	0.11 (0.02–0.58)
NIHSS score at 14 days,median (IQR)	2 (1,6)	15 (12,42)	< 0.001	
mRS(0–2) at 14 days,n(%)	23 (63.9)	2 (8.7)	< 0.001	18.6 (3.7–92.2)
mRS at 14 days,median (IQR)	2 (1–3)	5 (4–6)	< 0.001	
mRS(0–2) at 90 days,n(%)	26 (72.2)	2 (8.7)	< 0.001	27.3 (5.38–138.4)
mRS at 90 days,median (IQR)	1 (0.25–3.75)	5 (4–6)	< 0.001	

Note: *sICH* symptomatic intracranial haemorrhages, *OR* odds ratio, *CI* confidence interval, *NIHSS* National Institute of Health Stroke Scale, *mRS* modified Rankin Scale

### Prognosis

In a univariate analysis, the following variables were identified as predictors of a good outcome: young age (age ≤ 80 years), low NIHSS before thrombectomy (NIHSS≤14), small core infarct volume on preintervention CTP (core volume ≤ 50 ml) and LSA+ (Tables 3). In a multivariate analysis, a small core infarct volume (OR 6.74,95% CI 1.148–39.569, *P* = 0.035) and LSA+ (OR 22.114,95% CI 3.339–146.470, *P* = 0.001) were significant independent predictors of good outcome at 3 months (Table 3).

**Table 3:** Univariate and multivariate analysis of Determinants of a good outcome

Covariate	Unadjusted OR	95%CI	*p* value	Adjusted OR	95%CI	*p* value
Mismatch ratio ≥ 1.8	2.000	0.45–8.90	0.290			
Good collateral circulation	2.017	0.63–6.46	0.234			
Onset to clot first reperfusion time ≤ 6 h	0.635	0.22–1.84	0.401			
Successful recanalization (TICI≥2b)	1.603	0.35–7.42	0.413			
Age ≤ 80 years	3.409	0.82–14.20	0.081	3.275	0.49–21.84	0.220
NIHSS≤14 before thrombectomy	5.556	1.74–17.79	0.003	2.377	0.49–11.55	0.283
sICH	0.122	0.02–0.61	0.006	0.550	0.07–4.33	0.570
Core infarct volume ≤ 50 ml	6.863	1.71–27.58	0.004	6.740	1.15–39.57	0.035
LSA+	27.300	5.38–138.42	< 0.001	21.589	3.32–140.47	0.001

Note: *OR* odds ratio, *CI* confidence interval, *NIHSS* National Institute of Health Stroke Scale, *sICH* symptomatic intracranial haemorrhages

## Discussion

This study showed that except the core infarct volume, the appearance of lateral LSAs on pre-intervention DSA was independently associated with good functional outcome at 90 days after thrombectomy in patients with acute M1 segment of MCAO. Several studies have evaluated the correlation between the appearance of LSAs and basal ganglia infarction after thrombectomy. Kleine et al. [16] reported that LSAs occlusion patterns predicted infarction in associated striatal subterritories with a positive predictive value of 96%. Loh Y et al. [17] discovered that preintervention diffusion MRI evidence of injury in the basal ganglia before thrombectomy predicts poor outcome including worse dysfunction and disability at discharge, longer hospital stays, and higher rates of hemorrhage after intervention. Friedrich et al. [18]reported that the distance from the carotid T to thrombus in acute middle cerebral artery stroke independently predicts basal ganglia infarction after mechanical thrombectomy with high sensitivity and specificity. However, the correlation between the appearance of LSAs and the prognosis of thrombectomy is neglected. Our study reported their correlation and provided a simple and new thought for the decision making of thrombectomy.

It is supposed that patients with LSA+ before intervention would have a better clinical outcome for several reasons. (1) Although ischemic penumbra is reversible with early reperfusion, deep brain tissue like basal ganglia and internal capsule which are responsible for the transmission of key neural fiber pathway mainly depends on perforator arteries for blood supply, which have poor collateral circulation [19]^.^ Once the LSAs are blocked, irreversible ischemic lesions are formed and even with quick and successful revascularization the functional deficits might not reversible [20]. (2) It is reported that regional ischemic vulnerability of the brain differs between the cortex and basal ganglia, the tolerance of cortex to ischemia is better than that of internal capsule. This could contribute to the futile recanalization phenomenon, defined as the lack of a clinical benefit despite angiographic recanalization [21]. Kaesmacher J et al. [22] found that the appearance of LSAs on Magnetic Resonance Angiography after thrombectomy was associated with good outcome. Our results are similar to theirs, but more focused on the assessment of LSAs before thrombectomy, which might be helpful to guide clinical decisions and prognosis. (3) Hemorrhagic transformation is prone to occur after reperfusion treatment in deep brain tissue supplied by LSAs without collateral flow. In this study, the LSA+ group had a lower risk of sICH (8.3% versus 47.8%) compared to the LSA- group. A recent research reported that postoperative hemorrhagic transformation was detected in 25 of 55 (45.5%) patients after successful endovascular recanalization for acute ischemic stroke with large vessel occlusion involving the LSAs [23].

The study of independent factors determining the effect of thrombectomy is a hot topic of concern. The existing evidence showed that time to treatment, infarct core volume and collateral circulation may be used as reference factors. In particular, the DAWN trial [24]and DEFUSE-3 trial [25] suggests that the value of perfusion imaging is more prominent. However, multimodal imaging is not widely used. Thus, we are still in lack of concise imaging signs which are intuitive and easy to popularize. Using DSA to evaluate LSAs has several advantages in clinical application and promotion. First, this imaging marker is routine examined before thrombectomy. In addition, compared with preoperative multi-model imaging (CT or Magnetic resonance), using DSA to evaluate LSAs has advantages of shorter time and easier operation. Current research showed that it is feasible to assess LSAs using 3 T intracranial T1-weighted vessel wall imaging (VWI) or 7 T TOF-MRA [26, 27]. However, LSAs are generally not visible in conventional MRA, and VWI or 7 T MR angiography is difficult to be widely used in clinical practice. DSA is still considered as “gold standard” for observing the LSAs. The assessment of LSAs on pre-interventional DSA provides potentially new decision-making factors for emergency thrombectomy in hospitals without preoperative evaluation of core infarct and ischemic penumbra. This study also confirmed the core infarct volume was an independently prognostic predictors for thrombectomy, which is similar to other studies [28–30].

Our study had several limitations, including the retrospective design and limited sample size. There is potential bias in this retrospective study. Firstly, baseline data such as NIHSS score before thrombectomy was significantly different between the LSA+ and LSA- group. And the onset to puncture time in LSA+ group was longer than LSA- group although there were no statistical differences between the two groups. We used the multivariate analysis to adjust for these imbalances. Secondly, missing data is common and may cause bias in retrospective study. Our study was conducted in two advanced stroke centers with good clinical information collection and follow-up. Thirdly, because of the small sample size, the statistical power would be relatively low. Compared with LSA - group, LSA + group has higher recanalization rate, smaller ischemic core volume and lower NIHSS score before thrombectomy, which might affect the prognosis, so the correlation between LSA+ and outcome might be overestimated. In addition, the assessment of LSAs by DSA has limited spatial resolution. Two patients differed in their assessment mainly because of small LSAs not obvious and partial anatomical variations exists in LSAs. The two DSA reviewers are also thrombectomy surgeons. They participated in the initial endovascular intervention on 26 of 59 patients. Therefore, it is necessary to develop prospective research with more research sites and larger sample size to further verify results of the study.

## Conclusions

This study suggested that the appearance of LSAs on pre-interventional DSA would be potentially helpful for predicting good clinical outcome after stent-retriever thrombectomy in patients with acute M1 segment of MCAO. Our study provided a new thought for the patient selection for thrombectomy especially in those stroke centers without multimodal imaging.

## Data Availability

Study related data and materials are accessible on request from the corresponding author on reasonable request.

## Abbreviations

CBF: Cerebral blood flow
CI: Confidence interval
CT: Computed tomographic
CTA: Computed tomography angiography
CTP: Computed tomography perfusion imaging
DNT: Door-to needle time
DSA: Digital subtraction angiography
DT: Delay time
LAO: Large artery occlusion
LMCA: Left middle cerebral artery
LSAs: Lateral lenticulostriate arteries
LSA+: Appearing of lateral LSAs
LSA-: Sparing of lateral LSAs
MCAO: Middle cerebral artery occlusion
mRS: Modified Rankin Scale
mTICI: Modified thrombolysis in cerebral infarction
NIHSS: National Institute of Health Stroke Scale
OR: Odds Ratio
rLMC: The regional leptomeningeal score on CTA
rtPA: Recombinant tissue plasminogen activator
sICH: Symptomatic intracranial haemorrhages
